# Web-Based Medical Service: Technology Attractiveness, Medical Creditability, Information Source, and Behavior Intention

**DOI:** 10.2196/jmir.8114

**Published:** 2017-08-02

**Authors:** Shan Huei Wang

**Affiliations:** ^1^ Department and Graduate Institute of Business Administration National Taiwan University Taipei Taiwan

**Keywords:** web-based medical service, technology attractiveness, medical creditability, information source, behavior intention

## Abstract

**Background:**

Web-based medical service (WBMS), a cooperative relationship between medical service and Internet technology, has been called one of the most innovative services of the 21st century. However, its business promotion and implementation in the medical industry have neither been expected nor executed. Few studies have explored this phenomenon from the viewpoint of inexperienced patients.

**Objective:**

The primary goal of this study was to explore whether technology attractiveness, medical creditability, and diversified medical information sources could increase users’ behavior intention.

**Methods:**

This study explored the effectiveness of web-based medical service by using three situations to manipulate sources of medical information. A total of 150 questionnaires were collected from people who had never used WBMS before. Hierarchical regression was used to examine the mediation and moderated-mediation effects.

**Results:**

Perceived ease of use (*P*=.002) and perceived usefulness (*P*=.001) significantly enhance behavior intentions. Medical credibility is a mediator (*P*=.03), but the relationship does not significantly differ under diverse manipulative information channels (*P*=.39).

**Conclusions:**

Medical credibility could explain the extra variation between technology attractiveness and behavior intention, but not significant under different moderating effect of medical information sources.

## Introduction

With the increase in the aging population and rising Medicare expenditures, medical circles [1-3] and management practitioners are debating how people can use technology and the Internet to improve health services [4,5]. Web-based medical services (WBMS) such as eHealth, mHealth, and telehealth seem feasible solutions [6,7]. WBMS is considered as one of the most innovative services in medical technology in the 21^st^century [8]; however, promoting these services is still a challenge [5]. There are 3 impediments: perceived usefulness, behavior change, and medical law limitation.

Most WBMS focus on patients or potential patients with chronic conditions such as hypertension and diabetes. It is difficult for patients who do not use WBMS to see their value. The goal of WBMS is “prevention is better than cure.” That means WBMS needs to take a long time to show its effectiveness before serious symptoms occur. In addition, most patients are accustomed to face-to-face clinic services. Older patients in particular do not want to communicate with health care via the Internet. Finally, medical advertisement and doctor-endorsement on public are illegal for hospitals and related institutions, which makes word of mouth and noncommercial referrals by medical workers play an important role in WBMS.

A large body of literature has explored the relationship between the technology acceptance model and behavior intention in medical fields [2,7,9-13]. Results showed that perceived usefulness (PU) and perceived ease of use (PEOU) have positive impact on behavior intention. Other studies have investigated these relationships from the viewpoint of physicians and nurses [4,14-17]. Unlike other medical services, WBMS incorporates the interaction among medical workers and patients into the Web-based technology system to deliver medical services [18,19]. It is a patient-centered service designed to improve access to care for patients who need long term care or who have limited mobility [20], however, exploring from patient’s viewpoint is still a neglected area.

Earlier studies have explored antecedent factors [14,16,21], and moderating factors which affect technology attractiveness, in terms of PU, PEOU, and behavior intention [22]. Previous studies claim that medical credibility plays an important role in WBMS [20,23]; however, few studies explore the mediating effects of medical credibility on this relationship. In addition, under the constraints of medical law, different sources of medical information may result in different moderating effects among technology attractiveness, medical credibility and behavior intentions on WBMS [24,25]. The purpose of this study was, therefore, to explore the relationship among technology attractiveness, medical credibility, information source, and behavior intention on WBMS service. The objectives of this study were to (1) examine the technology attractiveness of WBMS and behavior intention from inexperienced patients’ view point, (2) examine the mediating effect of medical credibility, and (3) explore the moderated-mediation effect of different medical information sources.

This study begins with a review of the literature on WBMS, technology attractiveness, medical credibility, and medical information sources. Based on the literature review, this study formulates the hypotheses, describes the methods and sample, and presents the results. Finally, the theoretical contribution, managerial implications, and future directions for research are discussed.

### Literature Review and Hypotheses

#### Web-Based Medical Service Model

Web-based medical service (WBMS) is defined broadly as the use of Information and Communication Technologies (ICT) to provide medical information and services, including telephone intervention, medical education, and timely medical consultations [6]. It is called eHealth and mHealth (mobile health) in the Journal of Medical Internet Research (JMIR) and tele-health in other journals such as New England Journal Medicine (NEJM) and the Journal of the American Medical Association (JAMA). It is a patient-centered and online to offline service that provides patients with timely medical consultations at home and is a self-management medical service [1,26]. It is also a promising strategy for improving heart failure outcomes by monitoring patients remotely; therefore, physicians can intervene early if there is evidence of clinical deterioration [27].

The service process of WBMS is as follows: a patient participating in the service is given a set of specifically designed equipment, including a mini-personal computer or mobile phone and several devices to measure physiological indicators, which are installed at his or her home. Patients are requested to measure several physiological indicators (eg, blood pressure, blood sugar, ECG, and SPO2), and these indicators are automatically uploaded to the database of the hospital via the Internet. If some of the indicators are outside the acceptable ranges set by physicians according to each patient’s physical condition, nurses will then check relevant historical indicators and provide appropriate medical education and consultations immediately. If the patient’s condition is not improving, then an early doctor visit or emergency services may be necessary.

#### Technology Attractiveness and Behavior Intention

In this study, technology attractiveness is defined as the degree of users’ acceptance of new technology, in terms of PU and PEOU of WBMS. PU is referred as the degree to which a person believes that using a particular system would enhance his or her job performance, and PEOU is referred as the degree to which a person believes that using a particular system would be free of effort [7,9].

A number of studies have investigated the relationship between technology attractiveness and behavior intention of medical workers [4]. One stream focuses on physician’s intention [4,14,16,17]; the other focuses on nurse’s intention [14]. Wu et al [17] find that when making a decision to accept or reject a telemedicine technology, physicians appear to be fairly pragmatic, concentrating on the technology’s usefulness rather than on its ease of use. Similarly, Ammenwerth et al [14] introduced a computer-based nursing documentation system and systematically evaluated its preconditions and effects in a pretest-posttest intervention study. The results showed that the amount of nurse’s self-confidence when using computer is important before technology system implementation stage, but after implementation stage, the fit between nursing workflow and the functionality of the system were relatively more important. In sum, prior studies all support that PU and PEOU of computer and Internet access at workplace would be helpful in increasing medical workers’ behavior intention [15,17]. However, unlike other medical service, WBMS is a 3-sided cooperative model, including health care worker, patient, and technology. Patients’ acceptance will affect the success of WBMS in the early stages, but surveys from inexperienced patients’ viewpoints are still scarce [2,26,28]. According to the above reasons, this study proposes following hypothesis:

Hypothesis 1: There is a positive relationship between technology attractiveness and potential patient behavior intention.

#### The Mediation Effect of Medical Credibility

Previous studies focused on the antecedent factors of intention behavior (see the review article by Or and Karsh [21]), and moderating effects on technology attractiveness and behavior intention [22]. Or et al [29] summarize that technology attractiveness, subjective norm, and healthcare knowledge can predict most of the variance in patients’ acceptance of web-based self-management technology. Sun and Zhang [22] find that organizational, technological, and individual factors can moderate the relationship between technology attractiveness and behavior intention; however, few studies have examined the mediating effects on this relationship [9].

Ajzen and Fishbein [30] claim that a person who carries out a behavior intention is affected by subjective norm and attitude towards the behavior. However, attitudes do not fully mediate the relationship between technology attractiveness, in terms of PU and PEOU, on behavior intention [9]. That means that more significant factors such as medical credibility can mediate this relationship. However, studies have seldom noted this relationship. Medical credibility is defined as the willingness of patients to be vulnerable to the actions of medical service based on the expectation that the medical workers will perform a particular and trusted medical treatment important to them [31,32].

Unlike traditional medical service, WBMS requires the simultaneous cooperation of medical workers, patients, and the web-based technology system to deliver medical services [18,19]. However, most elderly patients are accustomed to face-to-face clinic services and they do not like communication over the Internet. McGrail et al [20] found that older patients are more likely to see a known medical provider, and patients from the lowest socioeconomic strata were the least likely to use a virtual medical service. Hence, earning patients’ trust and strengthening the medical creditability of WBMS in the service delivery process is important.

There are 2 stages in the medical delivery process of WBMS. In the first stage, patients encounter the technology and perceive its potential function, convenience, and fairness [33]. After the patients have used the technology for a while, the medical workers will provide a monthly report that documents each day’s physiological indicators, medical education, and suggestions offered by nurses and physicians. In the second stage, patients will evaluate the credibility of medical reports and the whole medical service delivery process of WBMS [28]. An earlier study supports that when patients have positive perception on medical credibility and trust this service, they are more willing to recommend it [34]. Hence, this study argues that a successful business model of WBMS needs both patients’ confidence in the web-based technology system and their trust in medical service they contact via the Internet every day.

Hypothesis 2: A medical creditability plays a mediator role between technology attractiveness and potential patient’s behavior intention.

#### The Moderated Role of Medical Information Sources

A person who engages in a particular behavior or produces an intention is affected by his or her subjective norm and attitude towards the behavior. Subjective norm is defined as the way in which individuals decide to take a behavioral intention when facing social pressure to accept the opinion, which may come from family, friends, supervisors, and colleagues [30]. Fishbein and Ajzen [18] point out that individual behavior, such as assessing a product or service, is subject to the amount of information the person has obtained. In addition, the key person (or information source) has an important role in an individual’s evaluation of information [35]. For example, studies have found that the way in which consumers choose and evaluate information on medicines can influence their attitudes to and use of those medicines [23,25]. Peterson et al [25] interviewed people who researched medicines online. Some of those participants preferred information provided by pharmaceutical companies, others preferred information from governments, organizations, and medical schools, and still others preferred word of mouth from other consumers who had already taken that medication. Koufopoulos et al [24] found that communities can have positive treatment outcomes, particularly for the management of chronic illness. Some researchers have concluded that patients trust physicians more than they trust online medical information, which they consult before talking with their physicians [23,36,37]. Accordingly, this study proposes that different information sources have different effects on the relationship among technology attractiveness, patients’ trust in medical credibility, and behavior intention. This study investigates whether information sources have a moderated-mediation effect by word of mouth of friends, recommendations from medical workers, and advertisements from a nonofficial medical company.

Hypothesis 3: Medical information sources have a moderated-mediation role between technology attractiveness and potential patient’s behavior intention.

## Methods

### Conceptual Framework

**Figure 1:**
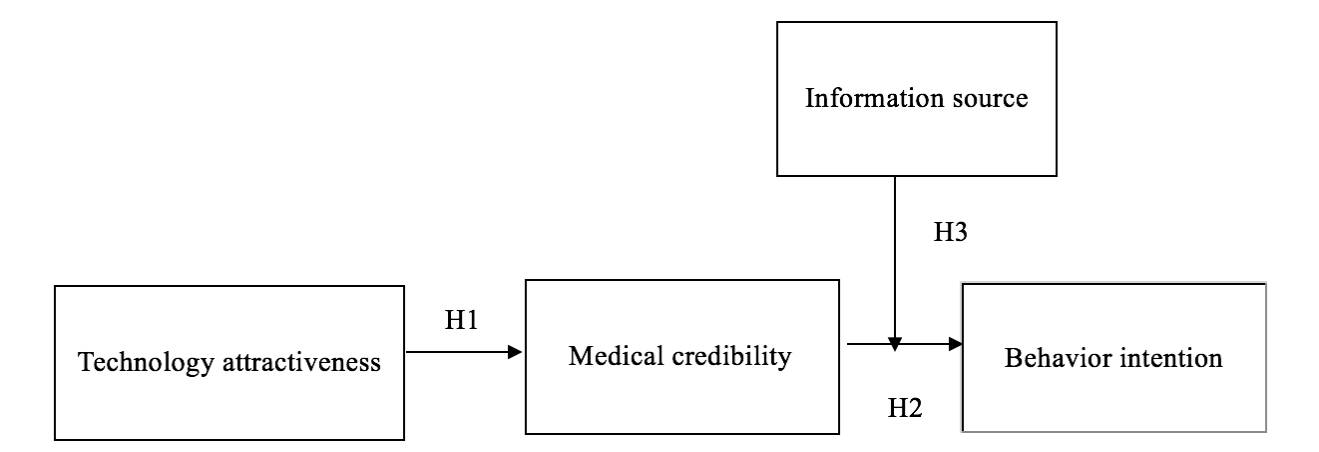
The framework of this study.

In order to explore (1) technology attractiveness of WBMS from potential patients’ viewpoint, (2) the mediate effects of medical credibility, and (3) the moderated-mediation effect of medical information sources, the framework of this study was constructed as shown in Figure 1.

### Sample Selection and Design

WBMS had been carried out for several years in Taiwan, but service providers wanted to know why it is still unfamiliar to and rejected by most of the population. This study collected 213 samples from several courses in Taipei; however, unlike previous studies, the purpose of this study was to investigate whether potential patients were willing to use or recommend WBMS to other potential patients. For this reason, the target sample had to meet the following criteria: (1) the participants had not used or heard of WBMS before the investigation; (2) they, their families, and friends were at high risk for high blood pressure, diabetes, and cardiovascular diseases; and (3) they had basic computer knowledge and Internet experience. Surveys with missing data were excluded, as were participants who did not meet the 3 criteria. Only 150 questionnaires were used in this study, 98 (65.3%) of which were completed by females and 52 (34.7%) were completed by males. The participants ranged in age from 26 to 50 years (mean 35). All participants used the Internet on an average of 3 to 4 times a week.

**Figure 2:**
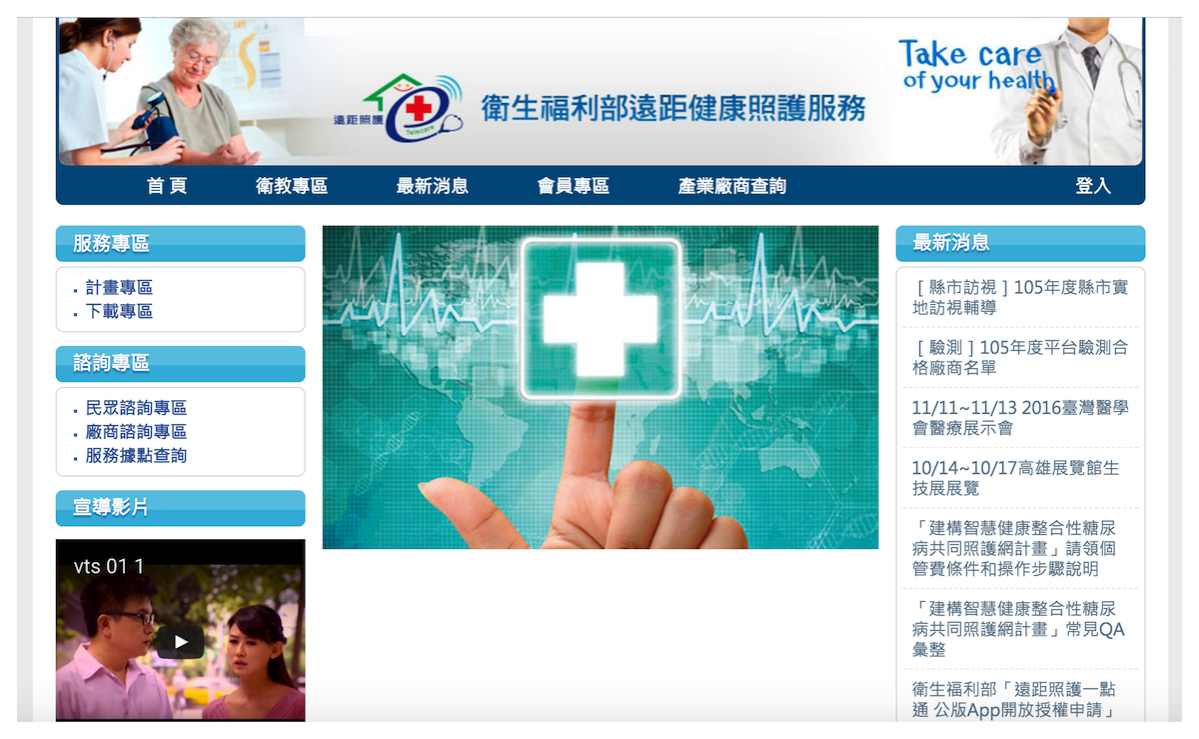
Web-based medical service.

To examine the effect of different information source, participants were randomly divided into 3 groups. Group 1 (n=50) manipulated information source from friends or family, group 2 (n=50) had an information source from nonofficial medical company, and group 3 (n=50) had information from an official medical source, such as a physician. Randomization was done by a random number generator [38]. In addition, all participants were asked to log into the eHealth website [39] that explained how to operate the WBMS instrument and the benefits of WBMS in Figure 2. This study conformed to the consolidated standards of reporting trials eHealth checklist (Multimedia Appendix 1) [40] and CHERRIES checklist to improve the quality of questionnaires checklist (Multimedia Appendix 2) [41].

### Measures

To explore the intention of potential patients when they used the different information source, this study combined experimental design and questionnaires to collect data through 3 kinds of different situations. It was not possible to find a perfectly suited questionnaire to measure our hypotheses, hence appropriate amendments mainly based on past scholars’ questionnaires were made. Furthermore, the adapted scales were originally in English, so previously established protocols of back translation were followed [42] to ensure that the translated Chinese questionnaire had similar meanings. Only 2 people who were bilingual in English and Chinese were hired, with one translating the English questionnaire into Chinese and the other cross-translating the items back into English. The researchers and the bilingual persons resolved any semantic inconsistencies and made minor adjustments in word choice before the survey was implemented. This study referred to Davis [9] questionnaire for testing technology attractiveness and behavior intention and followed Ajzen [35] and Gefen et al’s [19] questionnaires for the items of medical credibility. Participants rated all items on a 5-point Likert scale ranging from 1 (‘‘strongly disagree’’) to 5 (‘‘strongly agree’’). More details are shown in Multimedia Appendix 3.

### Data Analyses

This study used LISREL 8.54 (Scientific Software International, Inc. Skokie, IL, USA) to assess the convergent and discriminant validity of the constructs with a confirmatory factor analysis (CFA), and adopted maximum likelihood to rotate the constructs [43]. This study used multiple indexes to assess model fit: chi-square test (χ^2^), root mean of squared error of approximation (RMSEA), and its confidence interval, comparative fit index (CFI), incremental fit index (IFI), and goodness-of-fit index (GFI) [44]. In addition, scholars pointed out that the RMSEA higher than 1.0 meant poor model fit, between 0.05 and 0.08 meant accepted range, and lower than 0.05 meant good model fit [44]. CFI, GFI, and IFI were between 0 and 1, and the minimum acceptable value of CFI, GFI and IFI were 0.9 [45]. This study also used SPSS version 10 (SPSS Inc, Chicago, IL, USA) statistical software to examine the hypotheses. To test Hypotheses 1 and 2, this study followed Baron and Kenny [46] procedures for the partial mediation test. A total of 3 conditions were applied: (1) a significant effect of technology attractiveness on patients’ behavior intention, (2) a significant effect of technology attractiveness on medical credibility, and (3) a significant effect of medical credibility on potential patients’ intentions when model considered technology attractiveness.

To test the moderated-mediation effects in the model (Hypothesis 3), this study followed 3 condition steps recommended by Muller et al [47], (1) a significant effect of technology attractiveness on patients’ behavior intention, (2) a significant effect of technology attractiveness on medical credibility and a significant interaction between technology attractiveness and medical information source, and (3) a significant interaction between technology attractiveness and medical information source on potential patients’ behavior intention and a significant interaction between medical credibility and information sources.

## Results

Table 1 shows the means, standard deviations, and correlations of all dimensions. The correlations of 4 dimensions are highly relevant to each other, hence, this study used variance inflation factor (VIF) to examine the problem. None of the VIF was larger than 0.5, which means that there is no multicollinearity problem in this study [48].

### Reliability and Validity

To make sure the questionnaire items reflect the true meaning of constructs, a series of reliability and validity tests were conducted. This study used CFA to examine convergent validity, and 2 items were deleted in this study. The overall model fit of the measurement model was examined from several fit indexes: chi-square test was significant (χ^2^_51_=89.6, *P*<.001), RMSEA was .07, GFI was .91, CFI was .93, adjusted goodness-of-fit (AGFI) was .857, and all indicators ensured good model fit in the measurement [49-52]. Furthermore, the average variance extracted [20] of each construct was larger than 0.5, a good convergent validity in the measurement model [43,53]. Finally, Fornell and Larcker [53] claimed that a good discriminant validity was evident when the Average Variance Extracted (AVE) was larger than the square root of the construct correlation (Φ2). All correlations in Table 2 were significant (*P*<.001). All together, these results provided evidences for the convergent and discriminant validity of the proposed model. In addition, the range of composite reliability [23] was 0.79, indicating good internal reliability of the questionnaire items [54].

**Table 1:** Means, standard deviations, and intercorrelations of all dimensions. All correlation values are significant.

Variables	1	2	3	4	5	Means	Standard deviation
Perceived usefulness	1					3.91	0.82
Perceived ease to use	.47	1				3.81	0.74
Medical credibility	.47	.37	1			3.92	0.63
Willing to use	.34	.31	.37	1		3.84	0.95
Recommend to other person	.35	.34	.36	.55	1	3.90	0.74

**Table 2:** Confirmatory factor analysis standardized loading.

Variables		Standardized loading^a^	Average variance extracted	Composite reliability
**Perceived usefulness**			.5	.79
	PU1	.50		
	PU2	.47		
	PU3	.46		
	PU4	.70		
**Perceived ease to use**			.5	.79
	PEOU1	.64		
	PEOU 2	.68		
	PEOU 3	.93		
	PEOU4	.80		
**Medical credibility**			.5	.79
	PMP1	.73		
	PMP 2	.62		
	PMP 3	.61		
	PMP 4	.75		

^a^All standardized loading values are significant.

### Hypotheses Testing

This study used the recommendations of Baron and Kenny [46] and Muller et al [47] to examine the hypotheses. First, as reported in Model 1 of Tables 3 and 4, technology attractiveness were positively significantly related to potential patients’ behavior intentions, PU and PEOU were positively related to willingness to use (beta=.29, SE=0.14, *P*=.04; beta =.36, SE=0.11, *P*=.002) and to recommend to other persons (beta =.39, SE=0.12, *P*=.002; beta =.32, SE=0.09, *P*=.001), so Hypothesis 1 was supported. Second, PU and PEOU were positively significantly related to medical credibility (beta =.38, SE=0.08, *P*=.001; beta =.30, SE=0.06, *P*=.001) in Model 2. Third, after considering the technology attractiveness, medical creditability, and potential patients’ behavior intentions in Model 3, the coefficient of medical creditability was significant. Furthermore, PU and PEOU in Model 3 were significantly smaller than those in Model 1. Hence, medical credibility had partial mediating effect on technology attractiveness and potential patients’ behavior intentions, so Hypothesis 2 was supported. This study also used the Sobel test [55] to examine the mediating effect. Sobel test assumed that the relationships between the dependent variable and independent variables were hypothesized to have an indirect effect due to the influence of mediating effect. More specifically, in a regression model that includes the mediator, the effect of the independent variables is reduced, and the effect of the mediator is still statistically significant. The result showed that the medical credibility played a mediation role on the relationship between technology attractiveness and willingness to use (Z=2.32, SE=0.02, *P*=.03; Z=1.91, SE=0.04, *P*=.06) and the relationship between technology attractiveness and willingness to recommend (Z=1.96, SE=0.05, *P*=.05; Z=2.08, SE=0.03, *P*=.04). Hence, the Sobel test provided a more robust examination, and Hypothesis 2 was supported. Finally, this study explored whether medical information sources played a moderated-mediation effect. The testing conditions 1 and 2 were same as above; this study only described condition 3 here. Model 3 of Tables 3 and 4 showed that the interaction between medical credibility and medical information sources on willingness to use (beta =.01, SE=0.12, *P*=.39) and willingness to recommend to others (beta =.00, SE=.01, *P*=.39) were not significant, so Hypothesis 3 was not supported. Hence, medical information sources did not play a moderated-mediation role on technology attractiveness and potential patients’ behavior intentions when medical credibility existed.

**Table 3:** Hierarchical linear regression results for testing willing to use (mediating effect). Table entries corresponding to the predicting variables are unstandardized estimations of the fixed effects, two-tailed.

Variables^a^		Model 1	Model 2	Model 3
		Willing to use	Medical credibility	Willing to use
		beta (SE)	beta (SE)	beta (SE)
Constant		1.26 (0.53)	1.16 (0.29)	.87 (0.56)
**Independent variable**				
	Perceived usefulness	.29 (0.14)	.38 (0.08)	.16 (0.06)
	Perceived ease to use	.36 (0.11)	.30 (0.06)	.27 (0.13)
	Medical credibility			.30 (0.16)
	Information source x Medical credibility			.01 (0.12)
*r*^2^		.15	.38	.18

^a^n=150.

**Table 4:** Hierarchical linear regression results for testing recommend to others (mediating effect). Table entries corresponding to the predicting variables are unstandardized estimations of the fixed effects, two-tailed.

Variables^a^		Model 1	Model 2	Model 3
		Recommend to others	Medical credibility	Recommend to others
		beta (SE)	beta (SE)	beta (SE)
Constant		.99 (0.43)	1.16 (0.29)	.64 (0.45)
**Independent variables**				
	Perceived usefulness	.39 (0.12)	.38 (0.08)	.28 (0.13)
	Perceived ease to use	.32 (0.09)	.30 (0.06)	.23 (0.10)
	Medical credibility			.39 (0.12)
	Information source x Medical credibility			.00 (0.01)
*r*^2^		.24	.38	.28

^a^n=150.

## Discussion

### Principal Findings

The purpose of this study is to explore whether technology attractiveness, medical creditability, and different medical information sources could create different behavior intentions. The finding indicates that technology attractiveness must be significantly positive to enhance behavior intentions. In addition, medical credibility is a mediator but not significantly different under diverse manipulative information channels *.*

### Comparison With Prior Work

Prior studies explored technology attractiveness and behavior intention from the viewpoints of physicians and nurses. This study finds a positive relationship between technology attractiveness and potential patients’ behavior intentions. The result is completely consistent with Davis [9] TAM models. This finding indicates that even though potential patients have never used WBMS before, they are willing to share information about this medical service with their friends and family members, as they perceive the technology attractiveness of WBMS.

This study also finds that medical credibility plays a mediating role between technology attractiveness and patients’ behavior intentions. This finding echoes the conclusions of McGrail et al [20] and Hesse et al [23] that patients have more trust in the medical credibility of face-to-face clinic service than face-to-machine, meaning that medical credibility has an important role in the service delivery process of WBMS.

This study also finds that attitudes do not fully mediate the relationship between technology attractiveness and behavior intention [9]. This study provides additional evidence that medical credibility is an important mediator. Finally, this study finds that different sources of medical information do not significantly moderate the relationship. This finding is not consistent with previous studies that emphasize the effects of different information sources on behavior intention [23,35]. Possible reasons are that the dependent variables of this study are willingness to use and willingness to recommend. The target group of WBMS is patients with chronic medical conditions, such as cardiovascular disease, hypertension, and diabetes. According to social network theory, inexperienced patients may not need this service, but their friends and family may need it. It is not costly to share this information, so the information channel of WBMS may not be important for inexperienced patients after receiving the information.

### Conclusions

This study makes several contributions. Numerous studies have used the technology acceptance model (TAM) to explore customers’ intentions after new technology adoption, focusing on physicians’ and nurses’ intentions, but few studies have investigated the acceptance of web-based medical service by inexperienced patients. In addition, most extensive studies of TAM explore antecedent factors [14,16,21]. This study fills the research gap on mediation effect of TAM. The result shows that medical credibility is an important mediator in WBMS. Finally, this study is one of the few to explore whether differences in sources of medical information have a moderated-mediation effect on web-based medical service.

This study also offers some practical suggestions for telemedicine providers and hospitals wishing to promote WBMS. Patients are accustomed to face-to-face clinic services; they do not like communication via the Internet, especially if they are elderly. This study therefore suggests that telemedicine providers not only emphasize technology attractiveness but also the medical credibility of WBMS when introducing the service to new patients. WBMS combines online and offline services, and therefore, too much emphasis on the technology side of WBMS without taking into account the contributions of medical innovation and medical support may not appeal to the general population. This study suggests that the telemedicine providers and hospitals should pay attention to the convenience of technology (online) and medical credibility (offline) and educate patients on why prevention is better than treatment and why immediate medical intervention is important. Besides, most nonofficial medical companies think they are underperforming because they lack a physician’s recommendation. This study finds that the medical information channel is not a significant variable in influencing inexperienced patients’ behavior intentions. This study suggests that telemedicine providers should rethink their promotion strategy, rather than spend a lot of money on medical advertisement and doctor-endorsement fees. Finally, most of the target groups comprise patients with chronic disease. According to social network theory, the inexperienced patients may not need this service, but their friends and family may need the service. Telemedicine providers should not underestimate the power of social networks, especially the recommendations of inexperienced patients and the general population.

### Limitations

This study uses website descriptions and experiment designs to measure the intentions of inexperienced patients; however, the pay issues and different charging mechanisms are not considered, which could be one of the reasons that make the service difficult to promote. After all, there are still differences between intention and actual use behavior. This study suggests future research should consider this potential problem. Besides, medical information sources such as government promotion or community outreach service have not been considered. Hence, this study suggests further research should consider another effect of different sources.

## Appendix

Multimedia Appendix 1 CONSORT-EHEALTH checklist (V1.6.1).

Multimedia Appendix 2 Checklist for reporting results of Internet E-Surveys (CHERRIES).

Multimedia Appendix 3 Questionnaires.

## Abbreviations

AGFI: adjusted goodness-of-fit
AVE: Average Variance Extracted
CFA: confirmatory factor analysis
CFI: comparative fit index
GFI: goodness-of-fit
IFI: incremental fit index
JMIR: journal of medical Internet research
JAMA: journal of the American medical association
NEJM: New England journal medicine
PU: perceived usefulness
PEOU: perceived ease of use
RMSEA: root mean of squared error of approximation
TAM: technology acceptance model
VIF: variance inflation factor
WBMS: Web-based medical services
