# Supplementary material for: Web-Based Medical Service: Technology Attractiveness, Medical Creditability, Information Source, and Behavior Intention
Source: J Med Internet Res. 2017 Aug 2;19(8):e285. doi: 10.2196/jmir.8114 (PMC5559646; doi:10.2196/jmir.8114)
Supplement: Multimedia Appendix 2 [file jmir_v19i8e285_app2.pdf]

Multimedia Appendix_2: Checklist for Reporting Results of Internet E-Surveys (CHERRIES)

Item Category	Checklist Item	Explanation
Design	Describe survey design	Target population: Inexperienced patient of Web-based medical service(WBMS).<br>Survey topics: The primary goal of this study was to explore whether technology attractiveness, medical creditability and diversified medical information sources could increase users' behavior intention.<br>Survey design: This study explored the effectiveness of web-based medical service by using three situations to manipulate sources of medical information. This study referred to Davis (1989) questionnaire for testing technology attractiveness and behavior intention, and followed Ajzen (2002) and Gefen, Karahanna, Straub (2003) questionnaires for the items of medical credibility. Participants rated all items on a 5-point Likert scale ranging from 1 (“strongly disagree”) to 5 (“strongly agree”).
IRB approval Informed consent process	IRB approval	Not applicable. This study doesn't include Ethics issues.
	Informed consent	All participants were asked to log into the eHealth website (http://mohw.telecare.com.tw) that explained how to operate the WBMS instrument and the benefits of WBMS before filling the questionarries.
	Data protection	No personal identifying information was collected and record in the manuscript.
Development and pre-testing	Development and testing	To explore the intention of potential patients when they used the different information source, this study combined experimental design and questionnaires to collect data through three kinds of different situations. It was not possible to find a perfectly suited questionnaire to measure our hypotheses, hence we made the appropriate amendments mainly based on past scholars' questionnaires. This study referred to Davis (1989) questionnaire for testing technology attractiveness and behavior intention, and followed Ajzen (2002) and Gefen, Karahanna, Straub (2003) questionnaires for the items of medical credibility. Participants rated all items on a 5-point Likert scale ranging from 1 (“strongly disagree”) to 5 (“strongly agree”). The survey tool was translated into Chinese and back translated into English by bilingual speakers. The researchers and the bilingual persons resolved any semantic inconsistencies and

		made minor adjusts in word choice before the survey was implemented.
Recruitment process and description of the sample having access to the questionnaire	Open survey versus closed survey	The survey was open to participants who meet the eligibility criteria. (1) the participants had not used or heard of WBMS before the investigation, (2) they, their families and friends were at high risk for high blood pressure, diabetes, and cardiovascular diseases, and (3) they had basic computer knowledge and Internet experience. To complete the survey, participants had to log in the eHealth website that explained how to operate the WBMS instrument and the benefits of WBMS before filling the questionarries. Besides, to examine the effect of different information source, participants were randomly divided into three groups: friends/family, non-official medical company, and official medical institution
	Contact mode	All participants were asked to log into the eHealth website (http://mohw.telecare.com.tw) that explained how to operate the WBMS instrument and the benefits of WBMS before filling the questionarries.
	Advertising the survey	The survey was not advertised
Survey administration	Web/E-mail	The survey was web-based. All participants were asked to log into the eHealth website (http://mohw.telecare.com.tw) . To examine the effect of different information source, participants were randomly divided into three groups. Randomization was done by a random number generator (https://www.random.org)
	Context	This study context was based on the eHealth website. The different information sources was designed by prior studies.
	Mandatory/voluntary	The survey was voluntary. Participants were Randomly invited to the survey.
	Incentives	Survey completers were awarded a gift worth NT$20.
	Time/Date	Data were collected within one week in September 2012
	Randomization of items or questionnaires	To examine the effect of different information source, participants were randomly divided into three groups. Randomization was done by a random number generator (https://www.random.org)
	Adaptive questioning	Not applicable
	Number of items	The survey contained a short scenario description and 20 questions. Participants rated all items on a 5-point Likert scale ranging from 1 (“strongly disagree”) to 5 (“strongly agree”)..

	Number of screens (pages)	The total number of pages a participant could see was 78. Since participant may skip some page, the total number of pages would less than 78.
	Completeness check	All question items were required to be completed. Only completed surveys were included for analysis
	Review step	Participants were not required to review their responses at survey completion
Response rates	Unique site visitor	Not applicable
	View rate (Ratio unique site visitors/unique survey visitors)	All participants were asked to log into the eHealth website that explained how to operate the WBMS instrument and the benefits of WBMS (http://mohw.telecare.com.tw/) before filling the questionarries.
	Participation rate (Ratio unique survey Page visitors/agreed to participate)	Participants all agree to join the survey
	Completion rate (Ratio agreed to participate/finished survey)	This study collected 213 samples from several courses in Taipei. Surveys with missing data were excluded, as were participants who did not meet the three criteria. Only 150 questionnaires were used in this study. Survey response rate: 100%. Number of dropouts: 63; Number of completes:150
Preventing multiple entries from the same individual	Cookies used<br>IP check	Not used. This study combines both experiment design and questionnaire. All participants finished the questionnaire at the scene.
	Log file used<br>Registration	Not used.
Analysis	Handling of incomplete questionnaires	This study collected 213 samples from several courses in Taipei. Surveys with missing data were excluded, as were participants who did not meet the three criteria.
	Questionnaires submitted with an Atypical timestamp	This study collected the data from start to finish the questionnaire (Time Stamps).<br>The length of time required to complete the questionnaire varied widely across participants, average survey completion time was 20 minutes; Minimum was 15 minutes, hence this study did no set cut-offs point.
	Statistical correction	No statistical correction were used in the analysis section.
