# Supplementary material for: Web-Based Medical Service: Technology Attractiveness, Medical Creditability, Information Source, and Behavior Intention
Source: J Med Internet Res. 2017 Aug 2;19(8):e285. doi: 10.2196/jmir.8114 (PMC5559646; doi:10.2196/jmir.8114)
Supplement: Multimedia Appendix 3 [file jmir_v19i8e285_app3.pdf]

Question items used in this study

Constructs	Items	Measure	source
Perceived usefulness	PU1	Using WBMS can enhance my effectiveness in self-health management	Davis (1989)
	PU2	Using WBMS can improve self-health management of patients with cardiovascular diseases	
	PU3	I think WBMS can improve the quality of medical treatment	
	PU4	I though 24 hours medical consultation is useful	
Perceived ease of use	PEOU1	It is easy for me to become skillful in using WBMS	Davis (1989)
	PEOU2	Using WBMS can make self-health management easier	
	PEOU3	Measuring physical data and upload is a simple medical service	
	PEOU4	Although I do not use the WBMS before, but I think it is very simple	
Medical credibility	PMP1	I think WBMS is very professional, it diagnose heart disease by daily data	Ajzen (2002) and Gefen et al., (2003)
	PMP2	I trust and confidence on the professional service	
	PMP3	I think the instruments of WBMS service is accurately	
	PMP4	I think WBMS is a professional new-innovative medical service	
Behavior intention	BI 1	Listening to the commentary, I am willing to use.	Davis (1989)
	BI 2	Listening to the commentary, I am willing to recommend to other people.	
